# Structural basis for the functional properties of the P2X7 receptor for extracellular ATP

**DOI:** 10.1007/s11302-021-09790-x

**Published:** 2021-05-13

**Authors:** Lin-Hua Jiang, Emily A. Caseley, Steve P. Muench, Sébastien Roger

**Affiliations:** 1 School of Biomedical Sciences, Faculty of Biological Sciences, University of Leeds, Leeds, UK; 2 Faculty of Medicine and Health, Leeds Institute of Rheumatic and Musculoskeletal Medicine, St James’s University Hospital, Leeds, UK; 3 EA4245, Transplantation, Immunology and Inflammation, Faculty of Medicine, University of Tours, Tours, France

**Keywords:** Agonist binding, Receptor activation, Ion permeation, Large pore formation, Antagonism

## Abstract

The P2X7 receptor, originally known as the P2Z receptor due to its distinctive functional properties, has a structure characteristic of the ATP-gated ion channel P2X receptor family. The P2X7 receptor is an important mediator of ATP-induced purinergic signalling and is involved the pathogenesis of numerous conditions as well as in the regulation of diverse physiological functions. Functional characterisations, in conjunction with site-directed mutagenesis, molecular modelling, and, recently, structural determination, have provided significant insights into the structure–function relationships of the P2X7 receptor. This review discusses the current understanding of the structural basis for the functional properties of the P2X7 receptor.

## Introduction

The concept of P2 purinergic receptors for extracellular ATP (and ADP), when proposed by Burnstock, the pioneer of purinergic signalling, was met with extraordinary resistance [1]. Today, it is firmly established that ATP-induced purinergic signalling, mediated by two structurally and functionally distinctive subfamilies of P2 purinergic receptors known as ligand-gated ion channel P2X receptors and G-protein-coupled P2Y receptors, is a widely used signalling mechanism [2]. The P2X7 receptor was initially named the P2Z receptor due to its unique functional properties; receptor activation requires > 100 μM ATP, two orders of magnitude higher than concentrations required for activation of other P2 receptors, and prolonged receptor activation induces membrane permeabilisation and eventually cell death, earning its name of the cytolytic receptor [3, 4]. When the receptor manifesting these hallmark properties was molecularly identified, it was found to have a high degree of sequence homology with the six ion channel-forming P2X receptors (P2X1–P2X6), in addition to the same membrane topology consisting of intracellular N- and C-termini and two transmembrane domains linked by a large extracellular domain, albeit with an extended C-terminus. It was therefore united with the P2X receptor family as the seventh member [5–7]. The extensive research over the past decades, aided by P2X7-deficient mice and P2X7-specific antagonists, has considerably advanced our understanding of P2X7 receptor function in mammalian cells. The P2X7 receptor plays a critical role in mediating immune responses, bone remodelling, neuron-glia communication, and other physiological processes, and is also important in the pathogenesis of many conditions, including autoimmune diseases, neurodegenerative diseases, mood disorders, and cancers, making it an attractive therapeutic target (e.g. [8–24]).

The structure–function relationships of the P2X7 receptor were mostly inferred from mutagenesis and functional studies of other P2X receptors [25], but have recently been revolutionised by progress in the structural biology. The breakthroughs in determining the structures of the zebrafish P2X4 receptor in apo and ATP-bound states [26, 27] allowed us for the first time to directly see how ATP binds to the P2X receptor and opens the ion channel. Structures of P2X7 receptors have been determined in recent years, including the intracellularly truncated chicken and giant panda P2X7 receptors [28, 29], and notably the full-length rat P2X7 receptor [30]. Increased structural information offers an exciting opportunity to understand in molecular detail how the P2X7 receptor operates. In this review, we discuss structural and functional studies of P2X7 receptors and provide an overview of the current understanding of the structural basis for the functional properties of this physiologically and therapeutically important receptor.

## The overall receptor architecture

**Fig. 1:**
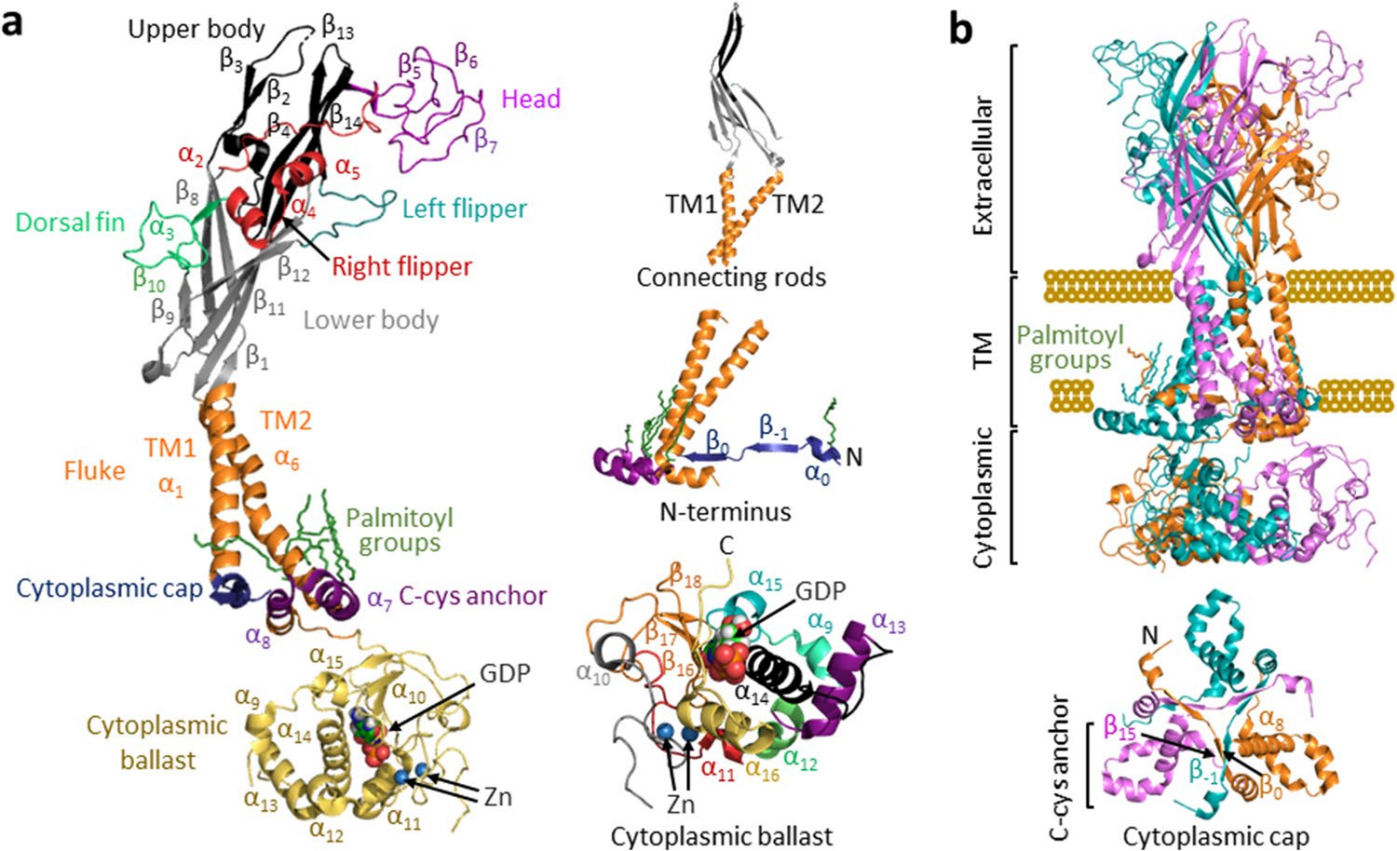
The P2X7 receptor architecture. **a** The secondary structure of single subunit in the rat P2X7 receptor in the apo state. The transmembrane domains (TM1 and TM2) are α-helical (α_1_ and α_6_, respectively), and the extracellular domain contains fourteen β strands (β_1_–β_14_) and four α-helices (α_2_–α_5_). The shape of the extracellular and transmembrane domains together resembles a leaping dolphin. Inserts shown in the right panel: the connecting rods that are formed by multiple anti-parallel β-strands in the upper and lower bodies and link the extracellular domain to the TM1 and TM2 domains (top); the N-terminus that is shown to contain one α-helix (α_0_) and two β-strands (β_−1_ and β_0_) (middle); and the C-terminus, excluding the missing segment (S443-R469), that encompasses ten α-helices (α_7_–α_16_) and four β strands (β_15_–β_18_) (bottom). The distal C-terminus in each subunit, including α_9_–α_16_ and β_16_–β_18_, constitutes the cytoplasmic ballast that contains one GDP-bind site and two Zn^2+^-binding sites. **b** The quaternary structure of the trimeric rat P2X7 receptor in the apo and closed state, with each subunit highlighted in different colour. Three extracellular domains are intervened with each other to form the extracellular part. The transmembrane part includes six α-helices, with the three TM2 (α_6_) α-helices lining the ion-permeating pathway in the centre and the three TM1 (α_1_) α-helices on the periphery. The cytoplasmic ballast rests beneath the transmembrane domains of a neighbouring subunit. Insert in the lower panel: the β_−1_ and β_0_ in the N-terminus of two adjacent subunits, together with β_15_ and α_8_ in the proximal C-terminus of the third subunit, forms the cytoplasmic cap. The cysteine-rich domain, linking the TM2 α-helix to the cytoplasmic cap, is hinged to the plasma membrane as the C-cys anchor via palmitoyl groups

The transmembrane and extracellular domains of P2X receptors, as anticipated from their high sequence homology, have similar secondary and quaternary structural arrangements, which have been borne out by receptor structures. As illustrated in Fig. 1a for the rat P2X7 receptor, both transmembrane domains (TM1 and TM2) are α-helical (α_1_ and α_6_, respectively) and the extracellular domain contains fourteen β-strands (β_1_–β_14_) and four α-helices (α_2_–α_5_). The overall structure of the transmembrane and extracellular domains of the receptor subunit resembles the shape of a leaping dolphin, with the extracellular domain making the main body, including the head, upper body, lower body, left flipper, right flipper, and dorsal fin, and the transmembrane domains the fluke (Fig. 1a). Multiple anti-parallel β-strands, including β_1_, β_2_, β_8_, β_9, _β_11 _and β_12_ in the lower body and β_13 _and β_14_ in the upper body, form rigid structures or the so-called connecting rods [25] that link the extracellular domain to the TM1 and TM2 α-helices, respectively (Fig. 1a). The intracellular domains of the P2X receptors, particularly the C-terminus, noticeably differ in length and exhibit low sequence similarity. In the rat P2X7 receptor, the N-terminus has one α-helix (α_0_) and two β-strands (β_−1_ and β_0_) and the C-terminus, excluding the segment missing in the structure (Ser^443^-Arg^469^ in the apo state or Ser^443^-Arg^471^ in the ATP-bound state), encompasses further ten α-helices (α_7_–α_16_) and four β-strands (β_15_–β_18_) (Fig. 1a).

The P2X receptor is a trimer, differing from other extracellular ligand-gated ion channels such as the tetrameric ionotropic glutamate receptor and the pentameric nicotinic acetylcholine receptor [31]. The homo-trimeric rat P2X7 receptor consists of extracellular, transmembrane, and cytoplasmic parts [30] (Fig. 1b). In the extracellular part, three extracellular domains are intimately intervened with each other via extensive inter-subunit and intra-subunit interactions and form the ligand-binding sites. The transmembrane part includes six α-helices, with the three TM2 α-helices lining the ion-permeating pathway in the centre of the receptor and the three TM1 α-helices on the periphery. There are three separate structural components in the cytoplasmic part [30]. Two β-strands in the N-terminus, β_−1_ from one subunit, and β_0_ from the other, together with β_15_ and α_8_ in the proximal C-terminus of the third subunit, form a cytoplasmic cap [30] (Fig. 1b). A similar structural component has been identified in the human P2X3 receptor containing intracellular membrane-juxtaposing domains [32]. The rat P2X7 receptor structures reveal palmitoylation of several residues, including Cys^4^ in the N-terminus and Ser^360^, Cys^362^, Ser^363^, Cys^374^, and Cys^377^ in the P2X7-specific cysteine-rich domain (CRD) in the proximal C-terminus, named the C-cys anchor [30] (Fig. 1a). This domain linking the TM2 to the cytoplasmic cap anchors both of them to the plasma membrane, functioning as a hinge. Finally, the distal C-terminus in each subunit, including β_16_–β_18_ and α_9_–α_16_, forms a ballast-like structure resting beneath the transmembrane domains of the neighbouring subunit [30] (Fig. 1a, b). Intriguingly, the cytoplasmic ballast contains a GDP-binding site and two Zn^2+^-binding sites (Fig. 1a) but their functional and physiological role remains to be established.

## The extracellular inter-subunit ATP-binding pocket

**Fig. 2:**
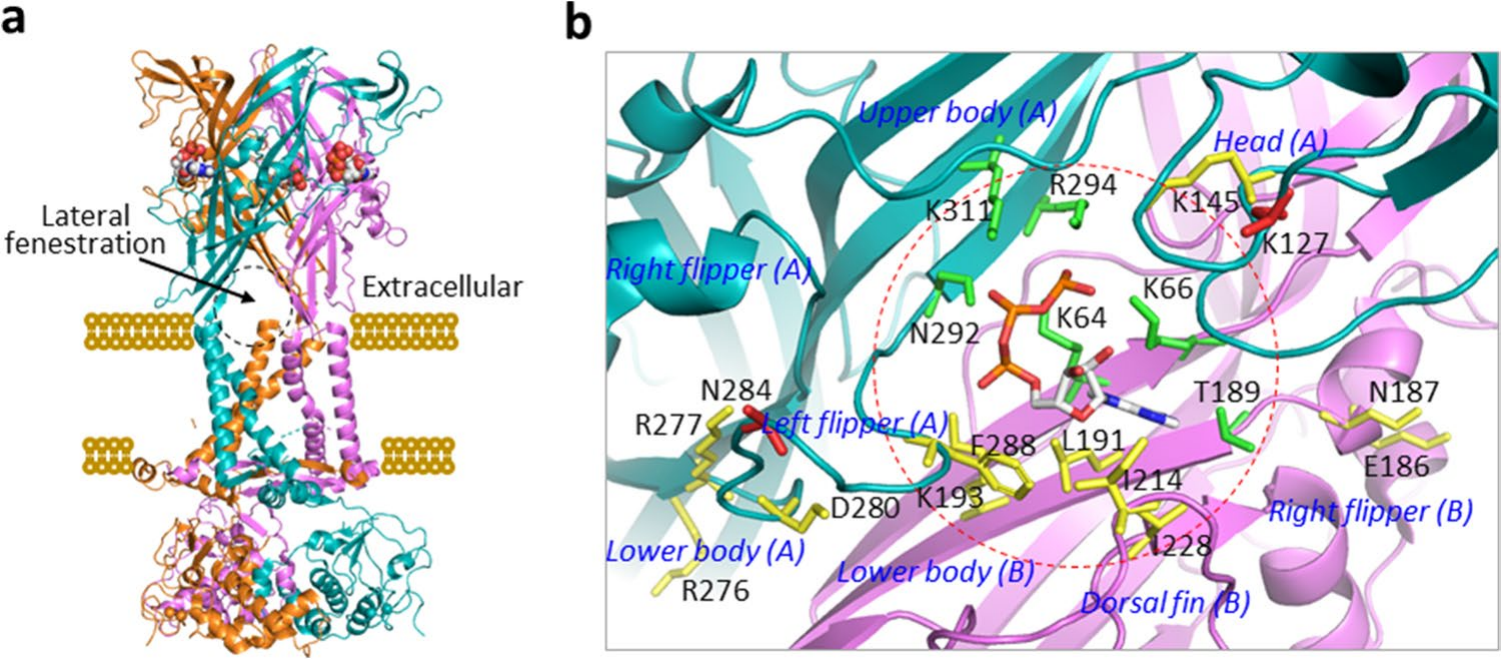
The extracellular inter-subunit ATP-binding pocket. (**a**) The quaternary structure of the rat P2X7 receptor in the ATP-bound and open state. ATP binds to the three subunit interfaces, inducing formation of three lateral fenestrations above the membrane as indicated by the dotted circle. (**b**) ATP binds at the apex of this inter-subunit pocket in the rat P2X7 receptor, formed by the head, upper body, and left flipper of one subunit (A) and the lower body and dorsal fin of the adjacent subunit (B). ATP binding is coordinated by K64, K66, and T189 in the lower body and N292, R294, and K311 in the upper body. The hydroxyl groups at positions 2′ and 3′ of the ribose are outward-facing. L191, K193, R276, and R277 in the lower body, I214 and I228 in the dorsal fin, K145 in the head, D280 and F288 in the left flipper, and E186 and N187 in the right flipper in the rat P2X7 receptor contribute to determining ATP sensitivity. K127 in the head and N284 in the left flipper are responsible for the greater agonist sensitivity of the rat P2X7 receptor than the mouse P2X7 receptor

Earlier studies analysing the single channel activation kinetics [33] and the ATP concentration-current response curves [34] suggest that the P2X receptor contains three ATP-binding sites. Extensive mutagenesis studies of mammalian P2X receptors, particularly the P2X1, P2X2, and P2X4 receptors, identified a subset of conserved residues in the extracellular domain that are crucial for receptor activation, including six hydrophilic residues corresponding to Lys^64^, Lys^66^, Thr^189^, Asn^292^, Arg^294^, and Lys^311^ in the rat P2X7 receptor, and provide further evidence to suggest that ATP binding occurs at the interface between two subunits [25]. The ATP-bound structures of the zebrafish P2X4, human P2X3, and rat P2X7 receptors [27, 30, 32] consistently support these ATP-binding features and, moreover, delineate how these residues coordinate ATP binding. The inter-subunit ATP-binding pocket is made up by the head, upper body, and left flipper of one subunit, and the lower body and dorsal fin of the adjacent subunit, as illustrated for the rat P2X7 receptor (Fig. 2). ATP binds at the apex of this inter-subunit pocket, assuming a U-shaped configuration and interacting with Lys^64^, Lys^66^, and Thr^189^ in the lower body and Asn^292^, Arg^294^, and Lys^311^ in the upper body (Fig. 2b). More specifically, the γ-phosphate group interacts with Lys^66^, Arg^294^, and Lys^311^, the β-phosphate group with Asn^292^, and the adenine with Thr^189^. Lys^64^ is located in the centre of the three phosphate groups and forms hydrogen bonds with all of them, as does the corresponding Lys^70^ residue in the ATP-bound zebrafish P2X4 receptor structure, highlighting the importance of this lysine residue in the interaction of the receptor with ATP [27]. Alanine mutation of Lys^64^ in the rat P2X7 receptor or the corresponding lysine residue in other P2X receptors led to loss of receptor activation [35]. Notably, the corresponding residue in the chicken P2X7 receptor is threonine (Thr^64^) [29]. Thr^64^ is expected to interact with ATP, but it remains to be verified whether this is the case and whether such an interaction is important for receptor activation. In all ATP-bound P2X receptor structures, the hydroxyl groups at positions 2′ and 3′ of the ribose face outwardly, indicating that ATP analogues with a bulky moiety at these positions readily bind to the ATP-binding site. Consistently, 2′(3′)-O-(4-benzoylbenzoyl)ATP (BzATP) is known as a potent P2X receptor agonist. As discussed below, 2′,3′-O-(2,4,6-trinitrophenyl)ATP (TNP-ATP) binds to the ATP-binding site as a competitive antagonist.

There is functional and structural evidence to indicate that additional residues in the ATP-binding pocket contribute in determining ATP sensitivity of the P2X7 receptors or receptor activation, as shown in the rat P2X7 receptor (Fig. 2b). Arg^276^ and Arg^277^ are in the lower body and highly conserved in P2X receptors. In the mouse P2X7 receptor, the charge-neutralising mutation R276A increased the ATP sensitivity, and charge-conserving mutations R276K and R277K enhanced ATP-induced responses, without altering the ATP sensitivity [36, 37]. Similarly, the R276H mutation in the human P2X7 receptor, resulting from a non-synonymous single nucleotide polymorphism (NS-SNP), increased ATP-induced responses [38]. Asp^280^ in the left flipper is mainly present in P2X7 receptors. The charge-neutralising mutation D280A in the human P2X7 receptor led to increased ATP sensitivity [39]. Phe^288^ also in the left flipper is highly conserved in P2X7 receptors, except that it is replaced with another hydrophobic residue, Tyr^288^, in the human P2X7 receptor. This residue is equivalent to the hydrophilic Ser^275^ in the human P2X3 receptor, which interacts with the α-phosphate group [32]. Such a local change may contribute to the lower ability of ATP to bind to the P2X7 receptor. In the neighbouring subunit, Lys^193^ in the lower body is positioned close to ATP. This residue is highly conserved in P2X receptors, and the corresponding residue in the zebrafish P2X4 receptor, Lys^193^, interacts indirectly with the α-phosphate group of ATP [27]. Alanine mutation of the corresponding residue Lys^188^ in the rat P2X2 receptor led to dramatically reduced sensitivity to ATP [40]. The dorsal fin of P2X7 receptors is relatively shorter than that of other P2X receptors and lacks the hydrophobic residue corresponding to Leu^217^ in the zebrafish P2X4 receptor that interacts with the adenine moiety of ATP [27]. Three hydrophobic residues, Leu^191^ in the lower body and Ile^214^ and Ile^228^ in the dorsal fin, are oriented towards the ribose and adenine, and together generate a local hydrophobic milieu that may assists ATP binding in a similar manner as Leu^217^. Both Leu^191^ and Ile^228^ are conserved in P2X7 receptors and Ile^214^ is present in most of them. Consistently, L191P, another NS-SNP mutation in the human P2X7 receptor, impaired ATP-induced current and dye uptake responses [41]. Lys^145^ in the head is conserved in mammalian P2X7 receptors but replaced by asparagine in the chick P2X7 receptor. The charge-neutralising mutation K145A in the rat P2X7 receptor reduced its sensitivity to BzATP [42]. Finally, Glu^186^ and Asn^187^ in the right flipper, outside of the ATP-binding pocket, are highly conserved in P2X7 receptors, except that Asn^187^ is replaced with aspartate in the chicken P2X7 receptor. NS-SNP mutations in the human P2X7 receptor altering these residues affect receptor responses to agonist; E186K completely abolished ATP-induced responses [41] and N187D reduced the BzATP sensitivity [43]. Thus, residues in the regions surrounding the ATP-binding site, some being highly conserved in P2X receptors and others specific to P2X7 receptors, are important in determining the sensitivity of P2X7 receptors to ATP and BzATP, by directly supporting agonist binding to the ATP-binding site, or indirectly influencing their access to the ATP-binding site or agonist binding-induced conformational changes for receptor activation.

Mammalian P2X7 receptors exhibit species differences in agonist sensitivity, most noticeably with the rat receptor being more sensitive to ATP and BzATP than the human, mouse, and dog receptors [7, 44–46]. The mouse P2X7 receptor with the extracellular domain replaced with that of the rat P2X7 receptor displayed virtually the same agonist sensitivity as the rat P2X7 receptor, indicating the extracellular domain is critical [44]. Further characterisation of the mouse P2X7 receptor carrying individual residues changed to the corresponding residues in the rat P2X7 receptor identified Asn^284^, or Asn^284^ together with Lys^127^, as main determinants for the greater sensitivity of the rat P2X7 receptor to ATP and BzATP, respectively. Lys^127^ and Asn^284^, located in the head or left flipper respectively, oversee the ATP-binding pocket (Fig. 2b). In P2X7 receptors of other species, position 127 is occupied by alanine in mouse and chicken or threonine in other species, and Asn^284^ is also present in human, guinea pig, and rabbit but substituted with various residues in other species, consistent with a role for residues at these positions in impacting agonist access to the ATP-binding site in a species-dependent manner.

Therefore, a subset of highly conserved residues coordinate ATP binding at the apex of an inter-subunit ATP-binding pocket in the P2X7 receptor, as in other P2X receptors. Additional residues in the ATP-binding pocket contribute to determining the agonist sensitivity of P2X7 receptors. However, there is still lack of a full understanding of the P2X7 receptors that exhibit a much lower ATP sensitivity and also activation by ATP in the form of ATP^4−^ [4].

## Agonist-induced conformational changes in the extracellular and transmembrane domains

Comparisons of the structures of the zebrafish P2X4, human P2X3, and rat P2X7 receptors in apo and ATP-bound states have disclosed considerable conformational changes accompanying receptor activation, particularly in the extracellular and transmembrane domains [27, 30, 32], as illustrated in Figs. 1b and 2a for the rat P2X7 receptor. Agonist binding to the ATP-binding site triggers the ATP-binding pocket to tighten. More specifically, the interaction of the ribose and adenosine with hydrophobic residues, Ile^214^ and Ile^228^ in the rat P2X7 receptor, induces upward movement of the dorsal fin, and subsequent outward movement of the lower body that is connected to the dorsal fin. The interaction of Phe^288^ in the lower body of the rat P2X7 receptor with the ribose and adenosine may impact such an outward movement of the lower body. The conformational change in the lower body causes enlargement of three lateral fenestrations adjacent to the membrane, which allow ions to enter into, or exit from, the transmembrane ion-permeating pathway (Fig. 2a). The conformational changes in the extracellular domains eventually drive, via the connecting rods (Fig. 1a), relative movement of TM1 and TM2 α-helices and outward flexing of the TM2 α-helices leading to opening of the ion-permeating pathway. Consistently, mutation of residues such as Lys^49^ and Phe^322^ in the β-sheets connecting the TM1 and TM2 α-helices altered activation of the rat P2X7 receptor by BzATP [47].

While the structures reveal that the upper body remains overall relatively immobile, there is increasing functional evidence to support that conformational changes occur in the upper body during P2X7 receptor activation. Simultaneous cysteine substitutions of Lys^81^ and Val^304^ in the human P2X7 receptor hindered receptor activation that was reversed by treatment with the reducing agent dithiothreitol (DTT) [48], indicating that these two residues or positions are in close vicinity in the closed state and move apart distantly in the open state. Replacement of Val^87^ in the human P2X7 receptor with the corresponding isoleucine in the rat P2X7 receptor enhanced current responses to ATP and BzATP [49], suggesting that residue at this position influences the local conformational change differently in the rat and human receptors. Moreover, as discussed below, the structures of the giant panda P2X7 receptor reveal an allosteric antagonist-binding pocket formed by the upper body that closes during receptor activation [28].

## The transmembrane ion-permeating pathway

**Fig. 3:**
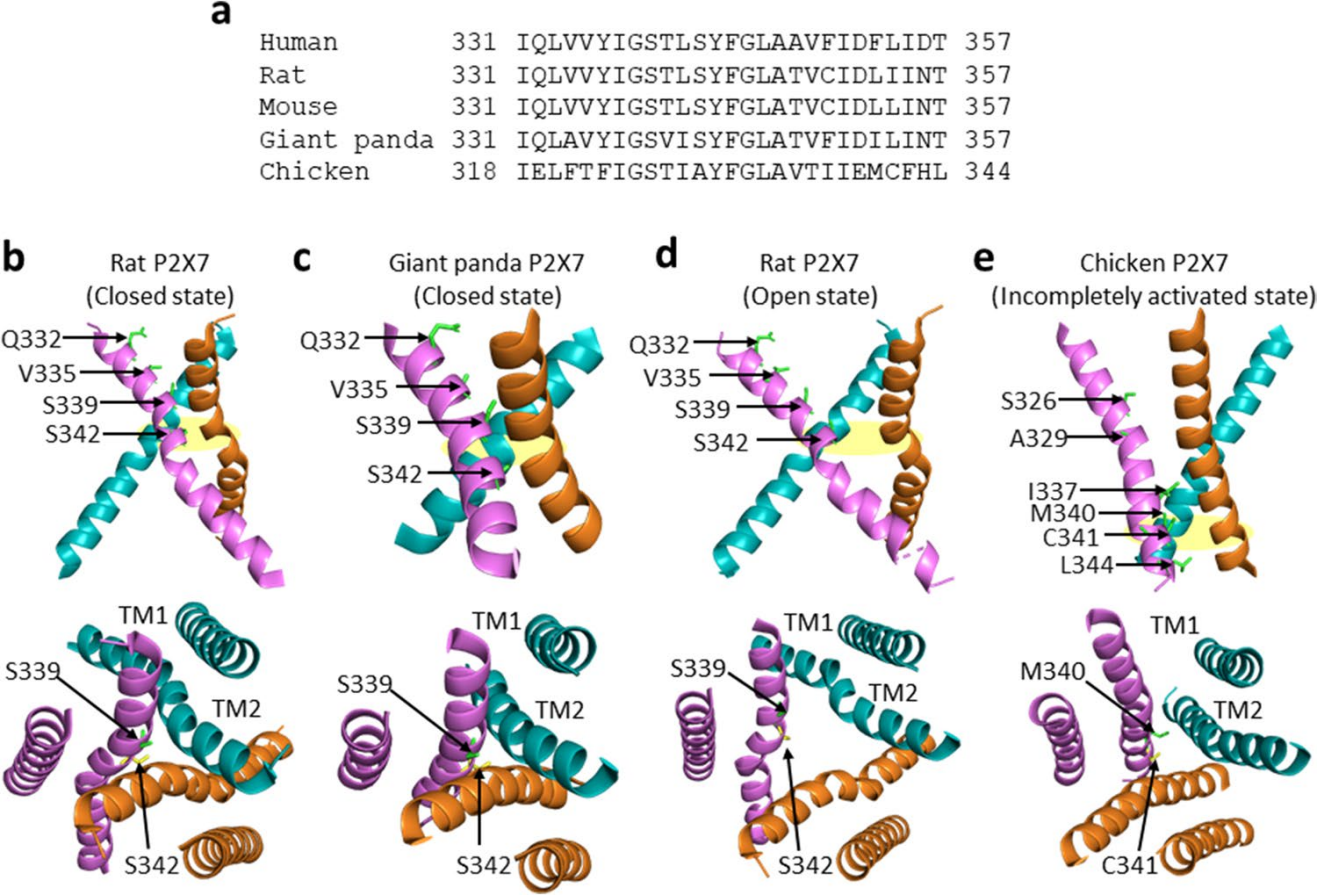
The transmembrane ion-permeating pathway. **a** Alignment of the TM2 sequences from human, rat, mouse, giant panda, and chicken P2X7 receptors. **b**–**c** The arrangements of the TM1 and TM2 α-helices in the rat (**b**) and giant panda (**c**) P2X7 receptor in the closed state, viewed from parallel to the membrane (top) or the intracellular side (bottom). Q332, V335, S339, and S342 in the extracellular part of the TM2 α-helix line the external vestibule in the closed state, with S339 and S342 located at the extracellular and intracellular ends of the ion-restricting gate. **d** The arrangements of the TM1 and TM2 α-helices in the rat P2X7 receptor in the ATP-bound and open state, viewed from parallel to the membrane (top) or the intracellular side (bottom). Movement of the TM2 α-helices apart from each other opens the ion-permeating pathway across the membrane with the pore at S342 remaining the narrowest part. The internal vestibule is wide and connected the cytoplasmic fenestrations between two TM2 α-helices, and permeant ions leave the internal vestibule via cytosolic fenestrations to reach the cytosol. **e** The arrangements of the TM1 and TM2 α-helices in the TNP-ATP bound chicken P2X7 receptor, viewed from parallel to the membrane (top) or the intracellular side (bottom). The internal vestibule is restricted at M340 and C341

One principal function of P2X receptors, including the P2X7 receptor, is to act as an ATP-gated cation-permeable channel. As mentioned above, the three TM2 α-helices constitute the transmembrane ion-permeating pathway in the centre of the receptor complex (Fig. 1b). In the apo structure of the rat P2X7 receptor, Asn^332^, Val^335^, Ser^339^, and Ser^342^ in the extracellular end of the TM2 α-helix (Fig. 3a) face the external vestibule, and Ser^339^ and Ser^342^ define the extracellular and intracellular boundaries, respectively, of the ion-occluding gate (Fig. 3b), at which the pore has a maximal radius of 0.1 Å [30]. The structures of the giant panda P2X7 receptor, determined in the antagonist-bound state, also reveal that the region between Ser^339^ and Ser^342^ forms the gate of the ion-permeating pathway in the closed state (Fig. 3c). Single channel recording in conjunction with the substituted cysteine accessibility method suggests that the gate of the human P2X7 receptor ion channel is located at the region surrounding Ser^342^ [50]. In the ATP-bound structure of the rat P2X7 receptor (Fig. 3d), the TM2 α-helices move apart from each other, opening the ion-permeating pathway continuously across the membrane with a minimal radius of 2.5 Å at Ser^342^, and Ser^339^ is rotated away from the centre of the external vestibule (Fig. 3d). In the human P2X7 receptor, simultaneous introduction of cysteine substitution at Asp^48^ and Ile^331^, located in the extracellular end of the TM1 and TM2 α-helices, respectively, prevented receptor activation, which was rescued by treatment with DTT [48]. Thus, these two residues or positions are in close proximity in the closed state and distance significantly from each other in the open state, supporting occurrence of considerable conformational changes in the external vestibule.

The apo and ATP-bound structures of the rat P2X7 receptor suggest that the intracellular part or the internal vestibule of the ion-permeating pathway remains widely open, even in the closed state, and is further connected to large spaces between two TM2 α-helices, named the cytoplasmic fenestrations [30]. It has been proposed that, in the open state, permeant ions leave the internal vestibule via the cytoplasmic fenestrations to reach the cytosol [30]. However, the structure of the chicken P2X7 receptor reveals noticeably different arrangements of the ion-permeating pathway, particularly the internal vestibule. This structure was determined with TNP-ATP bound to the ATP-binding site and is thought to be in an incompletely activated state, because the overall conformation of the extracellular domain more closely resembles that of the zebrafish P2X4 and human P2X3 receptors in the ATP-bound state rather than in the apo state [29]. The pore region surrounding Ser^326^ and Ala^329^ in the chicken P2X7 receptor (Fig. 3e), corresponding to Ser^339^ and Ser^342^ in the rat and giant panda P2X7 receptors (Fig. 3b–c), widely opens and is permissive to ion flow. In striking contrast with the exceptionally spacious intracellular part of the ion-permeating pathway in the rat P2X7 receptor structure (Fig. 3b), the internal vestibule lined by Ile^337^, Met^340^, Cys^341^, and Ler^344^ in the chicken P2X7 receptor, corresponding to Cys^350^, Leu^353^, Ile^354^, and Thr^357^ in the rat P2X7 receptor or Phe^350^, Phe^353^, Leu^354^, and Thr^357^ in the human P2X7 receptor (Fig. 3a), is highly restricted, with the pore radius being < 0.3 Å at Met^340^ and Cys^341^ (Fig. 3e). Such structural features strongly suggest that the internal vestibule plays a significant role in defining the ion channel function, which is indeed well supported by functional studies of the human and rat P2X7 receptors carrying mutations in the intracellular part of the TM2 α-helix [41, 51, 52]. Ala^348^ in the human P2X7 receptor is equivalent to Thr^348^ in the rat P2X7 receptor, corresponding to Val^335^ in the chicken P2X7 receptor (Fig. 3a). A348T, a NS-SNP mutation in the human P2X7 receptor, enhanced ATP-induced current responses, whereas introduction of reciprocal mutation T348A in the rat P2X7 receptor reduced, ATP-induced current responses [51]. Further characterisation of the human P2X7 mutant receptor with Ala^348^ replaced by different residues indicates that the current amplitude was overall inversely related to the size of the side chain of introduced residues, supporting the notion that the residue at this position considerably influences the ion flow through the internal vestibule. Replacement of Phe^353^ in the human P2X7 receptor with Leu^353^ in the rat P2X7 receptor also increased ATP-induced currents and, conversely, substitution of Asp^356^ in the human P2X7 receptor with Asn^356^ in the rat P2X7 receptor decreased ATP-induced currents. None of the mutations altered the agonist sensitivity [51]. These results also suggest that the different residues at positions 348 and 353, as well as at position 87 in the upper body discussed above, contribute to the different current responses of the human and rat P2X7 receptors. In addition, introduction of T348K, D352K, or D352N in the rat P2X7 receptor to add positive charges or neutralise existing negative charges at these positions conferred the resulting ion channel with measurable chloride permeability [52]. Collectively, functional studies provide evidence to support the importance of the intracellular part of the TM2 α-helix in determining the permeability of the P2X7 receptor ion channel. Evidently, further investigations are required to resolve the emerging discrepancies to achieve a clear understanding of the ion-permeating pathway in the P2X7 receptor in the closed and open states.

## The C-terminal cysteine-rich domain as an anchor to prevent receptor desensitisation

Agonist-induced current responses of P2X receptor ion channels exhibit remarkably different desensitisation kinetics. For example, the P2X1 and P2X3 receptors desensitise extremely fast, losing current responses within 1 s, and the P2X2, P2X4, and P2X5 receptors also desensitise but relatively slowly; however, the P2X7 receptor does not [53]. In addition, the P2X7 receptor exhibits striking sensitisation or facilitation, with the current responses increasing upon prolonged or repeated agonist exposure. There is evidence to support an important role for intracellular domains in receptor sensitisation or facilitation [39, 54–57]. Functional and structural studies consistently point to the P2X7-specific C-terminus, particularly the CRD, for its role in preventing receptor desensitisation. The human P2X7 receptor with deletion of the CRD (Cys^362^-Ala^379^) desensitised considerably, and, conversely, the human P2X1 or human P2X2 receptor with the CRD from the human P2X7 receptor inserted in the C-terminus exhibited substantially less desensitisation [39]. It is proposed, based on the structures of the human P2X3 receptor, particularly in the ATP-bound open and desensitisation states, that receptor desensitisation is associated with breakdown of the cytoplasmic cap and subsequent rearrangement of the TM2 α-helices. This may result in the closure of the ion-permeating pathway at Val^344^ in the human P2X3 receptor [32], which corresponds to Val^349^ in the rat P2X7 receptor or Thr^336^ in the chicken P2X7 receptor (Fig. 3a). As introduced above, the rat P2X7 receptor structures reveal that the P2X7-specific CRD, which connects the TM2 and the cytoplasmic cap, are palmitoylated at multiple residues [30]. As shown in the human P2X7 receptor [39], the rat P2X7 receptor with deletion of the CRD exhibited strong desensitisation [30]. Furthermore, there was considerable receptor desensitisation in the rat P2X7 receptor with simultaneous alanine substitution of all palmitoytable residues in the CRD (Ser^360^, Cys^362^, Cys^363^, Cys^371^, Cys^373^, Cys^374^, and Cys^377^). Taken together, these structural and mutational studies support the idea that the P2X7-specific CDR acts as the C-cys anchor to stabilise the cytoplasmic cap and TM2 α-helices and thereby prevent receptor desensitisation.

## The mechanistically enigmatic large pore formation

Prolonged or repeated receptor activation triggers formation of large pores across the plasma membrane that allow permeation of molecules of up to 900 Da. As described above, this functional property defines the P2X7 receptor, even though it has been reported that activation of some other P2X receptors can also induce large pore formation, albeit less consistently and profoundly. Large pore formation can be measured, most commonly by monitoring intracellular accumulation of extracellular positively charged fluorescent dyes that are considerably larger than small cations, such as YO-PRO-1 (378 Da) and ethidium (314 Da) [52]. Accumulating evidence supports the importance of this receptor functionality in ATP-induced P2X7-mediated regulation of physiological and pathological processes [59–63]. However, the mechanisms underlying large pore formation remain poorly defined [64, 65]. Attention has been drawn to the unique C-terminus of the P2X7 receptor. Indeed, the seminal study reporting the rat P2X7 receptor showed that truncation of the extra C-terminus in the P2X7 receptor abolished its ability to induce large pore formation without affecting its ability to form functional ion channel [5]. Similarly, the human P2X7B receptor, an alternative splice variant containing additional 18 residues inserted after Leu^346^ but without the last 249 residues in the C-terminus, functions as an ATP-gated ion channel, but its activation cannot induce dye uptake [66]. The mouse P2X7 receptor carrying Pro^451^ or Leu^451^ in the C-terminus shows virtually the same ion channel function, but only activation of the receptor harbouring Pro^451^, but not Leu^451^, can trigger large pore formation [60, 67]. Deletion of the CRD (Cys^362^-Val^380^) in the rat P2X7 receptor increased BzATP-induced dye uptake [68] but removal of the CRD (Cys^362^-Ala^379^) in the human P2X7 receptor inhibited BzATP-induced dye uptake [39]; the exact reason for such species-dependence remains unclear. Introduction of point mutations in the N-terminus in the human P2X7 receptor also impaired BzATP-induced dye uptake [39]. Thus, both the intracellular domains, particularly the C-terminus, can influence the ability of P2X7 receptors to induce large pore formation.

There is some evidence to suggest that the P2X7 receptor ion channel itself forms the large pore, and, furthermore, sustained receptor activation may trigger an increase in the size of the transmembrane ion-permeating pathway. Gly^345^ in the TM2 α-helix (Fig. 3a) is a completely conserved residue in P2X receptors that is thought to offer structural flexibility for conformal changes in the ion-permeating pathway. In the rat P2X7 receptor, tyrosine substitution of Gly^345^ abolished agonist-induced dye uptake, without affecting Ca^2+^ influx through the open ion channel [59], and alanine replacement resulted in significant effects on both current and YO-PRO-1 uptake responses [69]. Further supporting evidence was provided by examining the permeability of the rat P2X7 receptor ion channel [52]. Membrane potential is known to be a driving force for the flow of small cations through the open ion channel, and, similarly, YO-PRO-1 uptake exhibited strong voltage-dependence, becoming progressively less with the membrane potential shifting towards from negative to positive [52]. As discussed above, the study showed that the rat P2X7 receptor ion channel carrying T348K or D352N mutation in the intracellular end of the TM2 α-helix displayed measurable chloride permeability. These mutations also led to enhanced entry of negatively charged fluorescein isothiocyanate (387 Da) and reduced entry of positively charged ethidium. Moreover, exposure of the wide-type (WT), G345C, or T348C mutant receptor to 2-((biotinoyl)amino)ethyl methanethiosulfonate (MTSEA-biotin; 382 Da) in the closed state had no effect on ATP-induced currents and ethidium uptake [52]. There was also no effect from exposure of the WT receptor in the open state to MTES-biotin. However, MTSEA-biotin modification of introduced cysteines, when the mutant receptor was in the open state, reduced ATP-induced currents mediated by the G345C or T348C mutant receptor, and ATP-induced ethidium uptake shown at the G345C mutant receptor [52]. Similarly, when the WT receptor in the closed or open state or the G345C mutant receptor in the closed state were exposed to the neutral and larger MTSR (696 Da), there was no effect, and, in contrast, exposure of the G345C mutant receptor in the open state rapidly inhibited ATP-induced currents. These results support the concept that the P2X7 receptor ion channel in the open state can permeate nanometre-sized dyes, in addition to small cations [52].

Analysis of the human P2X7 receptor at single channel level indeed also supports that the P2X7 receptor ion channel is permeable to large organic cations, including N-methyl-D-glucamine (NMDG; 196 Da), with the permeability inversely related with the size of permeant cations but, importantly, there is no indication of change in the permeability during prolonged receptor activation [70]. Similarly, modification by methanethiosulfonates of introduced cysteines in the TM2 α-helix altered human P2X7 receptor single channel conductance and open probability, but such alterations remained unchanged during extended recording up to 30 min [50]. As discussed above, the ion-permeating pathway in the ATP-bound structure of the rat P2X7 receptor [30] is sufficiently wide to permit passage of small physiological cations but seems too narrow to permeate large organic cations or nanometre-sized fluorescent dyes. To conclude, the mechanisms for the large pore-inducing functionality of P2X7 receptors continue to remain enigmatic.

## The orthosteric TNP-ATP binding site

TNP-ATP is a synthetic ATP analogue with a trinitrophenyl group attached to the ribose moiety at 2′ and 3′ positions. An early study by whole-cell recording of agonist-induced currents demonstrated that TNT-ATP preferentially inhibits P2X1- or P2X3-contaning receptors with an IC_50_ of < 10 nM and may act as a non-competitive antagonist [71]. The same study also showed that TNT-ATP has a considerably low sensitivity for the rat P2X7 receptor (> 30 μM) [71], whereas a recent study reports that TNP-ATP more potently inhibits the chicken P2X7 receptor, with an IC_50_ of 3.6 μM [29]. Recent studies have determined the structures of the human P2X3 [32] and chicken P2X7 receptors [29] in the presence of TNP-ATP, revealing that TNP-ATP binds to the ATP-binding site in both receptors.

In the human P2X3 receptor, ATP binding is coordinated by Lys^63^, Lys^65^, Thr^172^, Asn^279^, Arg^281^, and Lys^299^ in the ATP-binding site, as described above by the corresponding residues, Lys^64^, Lys^66^, Thr^189^, Asn^292^, Arg^294^, and Lys^311^, in the rat P2X7 receptor (Fig. 2b), and in addition, by Ser^275^ in the left flipper, corresponding to Phe^288^ in the rat P2X7 receptor [32]. Namely, Lys^63^ interacts with the triphosphate groups, Lys^65^, Arg^281^, and Lys^299^ with the γ-phosphate group, Asn^279^ with the β-phosphate group, Ser^275^ with the α-phosphate group, and Thr^172^ with adenine. As anticipated, TNP-ATP interacts with the ATP-binding site via the ATP moiety, with the trinitrophenyl (TNP) moiety outward-facing. However, the ATP moiety adopts a distinct U-shaped configuration and forms different hydrogen bond networks. Asn^279^, Arg^281^, and Lys^299^ interact with the γ-phosphate group, Lys^65^ with the α-phosphate group, and Lys^63^ and Thr^172^ with the adenine. Furthermore, TNP-ATP interacts with Phe^174^ in the lower body, and, indirectly via Mg^2+^, with Asp^158^ in the head. These two residues are mainly present in the P2X1 and P2X3 subunits, suggesting their importance in defining high affinity binding of TNP-ATP to the P2X1- or P2X3-containing receptors [32].

**Fig. 4:**
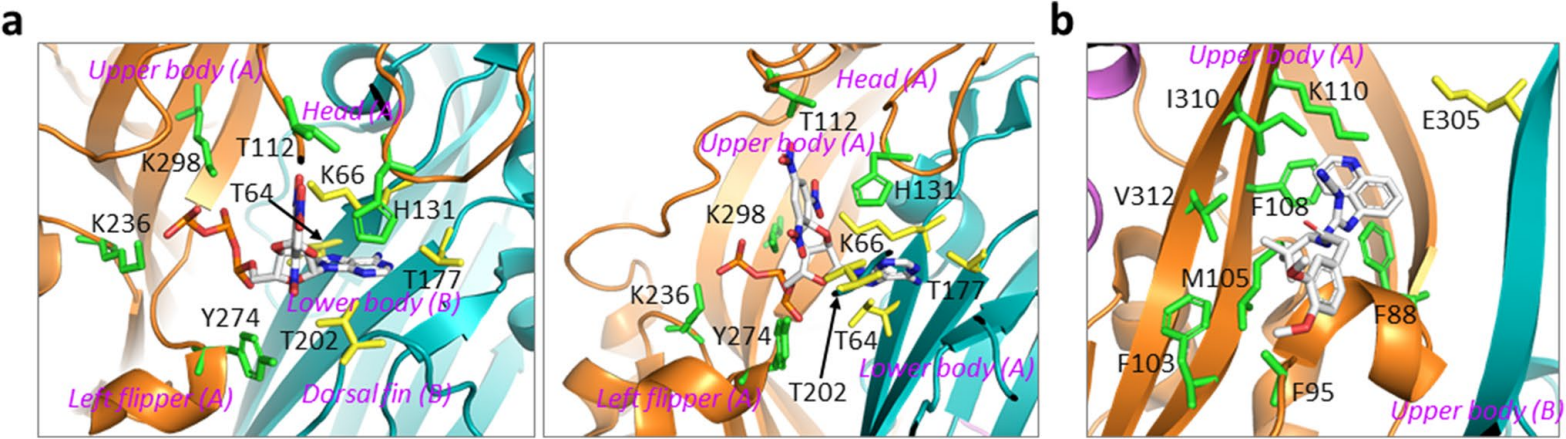
The orthosteric and allosteric antagonist-binding pockets. **a** TNP-ATP binding to the ATP-binding site in the chicken P2X7 receptor. The ATP moiety interacts with T64, K66, T177, and K298 in the ATP-binding site, and also K236 in the upper body. The TNP moiety interacts, through its three NO_2_ groups, with K66 in the lower body, T112 in the head, and T202 in the dorsal fin. H131 in the head and Y274 in the lower body are positioned close to TNP-ATP. **b** The allosteric binding pocket for chemically diverse P2X7 receptor antagonists in the giant panda P2X7 receptor, as illustrated for A740003 and the key hydrophobic residues that coordinate A740003 binding

The structure of the chicken P2X7 receptor show different interactions of TNP-ATP with the ATP-binding site [29]. Interestingly but not surprisingly, as introduced above, TNP-ATP binding induces significant conformational changes in the extracellular domain, which are nonetheless insufficient to open the ion-permeating pathway. As shown in Fig. 4a, in the chicken P2X7 receptor, the ATP moiety interacts with Thr^64^, Lys^66^, Thr^177^, and Lys^298^, corresponding to Lys^64^, Lys^66^, Thr^189^, and Lys^311^ in the ATP-binding site in the rat P2X7 receptor (Fig. 2b), and also with Lys^236^ in the upper body, equivalent to Asn^248^ in the rat and human P2X7 receptors. Thr^64^, Lys^66^, and Thr^177^ form hydrogen bonds with the adenine, Lys^236^ with the γ-phosphate group, and Lys^298^ with both the γ-phosphate and β-phosphate groups (Fig. 4a). Unlike in the human P2X3 receptor, the TNP moiety also participates in interactions with the chicken P2X7 receptor by forming hydrogen bonds between the three NO_2_ groups with Lys^66^ in the lower body, Thr^112^ in the head, and Thr^202^ in the dorsal fin, respectively. A threonine corresponding to Thr^112^ is also present in the human P2X7 receptor and replaced with serine in the rat P2X7 receptor, whereas the residue corresponding to Thr^202^ is variable, with isoleucine in the human and rat P2X7 receptors. In addition, His^131^ in the head and Tyr^274^ in the left flipper are positioned close to TNP-ATP. His^131^ is replaced with glutamine in the human and rat P2X7 receptors, and tyrosine corresponding to Tyr^274^ is also found in the human P2X7 receptor and substituted with phenylalanine (Phe^288^) in the rat P2X7 receptor. It is unclear, but interesting, to examine how these residues determine the difference in the sensitivity of the chicken and rat P2X7 receptors to TNP-ATP.

## The inter-subunit allosteric binding pocket for chemically diverse antagonists

Extraordinary efforts in medicinal chemistry driven by the interest in the P2X7 receptor as a therapeutic target have led to the discovery of numerous specific and potent P2X7 antagonists, with some exhibiting striking species differences [72–75]. Surprisingly, accumulating structural and functional evidence supports a common action mechanism for many of these chemically diverse antagonists via binding to the same inter-subunit allosteric binding pocket.

A74003, A804598, AZ10606120, and JNJ47965567 represent four structurally distinct mammalian P2X7 receptor antagonists. Competitive ligand binding analysis in a previous study suggests significantly overlapping binding of A804598 and JNJ47965567 [76]. GW791343 is an antagonist at the human and dog P2X7 receptors, but acts as a positive allosteric modulator at the rat P2X7 receptor [45, 46]. All of them are recently shown to be antagonists at the giant panda P2X7 receptor [28]. Structures of the giant panda P2X7 receptor, without the intracellular domains, in the presence of each of these antagonists reveal a common antagonist-binding pocket adjacent to the ATP-binding pocket, demonstrating allosteric and non-competitive antagonism. This allosteric antagonist-binding pocket is formed mainly by β_4_, β_13 _and β_14_ in the upper body (Fig. 1a) of two neighbouring subunits, as illustrated for A74003 binding to the giant panda P2X7 receptor (Fig. 4b). The pocket is lined by both hydrophobic and hydrophilic residues, including Phe^88^, Phe^95^, Phe^103^, Met^105^, Phe^108^, Lys^110^, Glu^305^, Ile^310^, and Val^312^. Alanine mutation of the hydrophobic but not the hydrophilic residues in the giant panda P2X7 receptor reduced the antagonist sensitivity (Table 1), suggesting that antagonist binding is mainly mediated by hydrophobic interactions. In addition, the effects of mutating individual hydrophobic residues depend strongly on individual antagonist (Table 1), reflecting their varying chemical structures and, thus, different interactions with residues in the allosteric binding pocket. These hydrophobic residues are either highly conserved or replaced by another hydrophobic residue in other mammalian P2X7 receptors; for example, Phe^95^ and Val^312^ are replaced by Leu^95^ and Ala^312^ in the rat P2X7 receptor, respectively. In the zebrafish P2X4 and human P2X3 receptors, the equivalent inter-subunit cavity formed by the β_13_ and β_14_ strands in the upper body also contains several hydrophobic residues, but the turret-like structure, corresponding to the antagonist-binding pocket in the giant panda P2X7 receptor, appears too narrow to accommodate even the smallest antagonist, A804598. These differences suggest that the size and shape of this allosteric binding pocket, as well as the interactions with P2X7-specific residues, are vital in determining the specific and high binding affinity binding of these antagonists to the giant panda P2X7 receptor [28]. This notion is further supported by P2X7-specific modification of cysteines introduced in the allosteric binding pocket [28]. Moreover, state-dependent cysteine modification of residues in the allosteric binding pocket in the giant panda P2X7 receptor suggests that the allosteric binding pocket is tightened during receptor activation. Such a finding has led to the proposal of a mechanism of antagonism for these P2X7 antagonists, in which occupancy of the allosteric binding pocket hinders the ATP binding-induced conformational changes in the upper body, which are required for receptor activation [28].

**Table 1:** Summary of mutational effects on the sensitivity of the giant panda and human P2X7 receptors to chemically diverse antagonists

Mutation	Position	A74003^a^	A74003^b^	A804598^a^	AZ10606120^a^	AZ10606120^b^	JNJ47965567^a^	GW791343^a^	A438079^b^	AZ11645373^b^
**K110**Y	Entrance	NS	NS	NS	NS	> 1.0^c^	~ 1.0	NS	NS	> 1.0^c^
**V304**C	Entrance	-	NS	-	-	< 1.0^c^	-	-	> 1.0^c^	-
E305A	Entrance	NS	NS	NS	NS	< 1.0^c^	NS	NS	< 1.0^c^	NS
**F88**A	Middle	> 1.0	> 1.0	> 1.0	NS	> 1.0	> 1.0	NS	~ 3.0	> 1.0
**D92**A	Middle	-	~ 2.0	-	-	~ 3.0	-	-	> 3.0	> 1.0
M105A	Middle	> 1.0	> 2.0	> 1.0	NS	~ 2.0	> 1.0	NS	NS	NS
**F108**C	Middle	NS	> 1.0	> 1.0	NS	NS	> 1.0	NS	1.0	< 1.0
**K297**G	Middle	-	< 1.0^c^	-	-	> 1.0^c^	-	-	> 1.0^c^	NS
**Y298**A	Middle	^−^	> 1.0	-	-	NS	-	-	< 1.0	NS
**I310**A	Middle	NS	NS	NS	NS	NS	NS	NS	< 1.0	> 1.0
**V312**A	Middle	< 1.0	< 1.0	NS	~ 1.0	NS	~ 1.0	< 1.0	< 1.0	< 1.0
**T90**V	Bottom	-	< 1.0	-	-	> 1.0	-	-	< 1.0	> 1.0
A91C	Bottom	-	> 1.0	-	-	NS	-	-	> 1.0	> 1.0
Y93A	Bottom	-	NS	-	-	< 1.0	-	-	< 1.0	< 1.0
T94V	Bottom	-	< 1.0	-	-	< 1.0	-	-	> 3.0	> 3.0
**F95**A	Bottom	NS	> 1.0	> 2.0	NS	NS	NS	-NS	> 3.0	> 3.0
P96A	Bottom	-	< 1.0	-	-	NS	-	-	> 1.0	> 3.0
**L97**P	Bottom	-	< 1.0	-	-	NS	-	-	< 1.0^c^	> 1.0
F103A	Bottom	< 1.0	< 1.0	> 1.0	> 1.0	> 1.0	> 1.0	> 1.0	> 3.0	> 1.0

Mutations of the allosteric binding pocket in the giant panda P2X7 receptor ^(a)^ or the human P2X7 receptor ^(b)^ reduce, or increase ^(c)^, the sensitivity to indicated antagonists. 1, 2, and 3 denote 10-, 100-, and 1000-fold change, respectively. *NS*, no significant change; -, not examined. Completely or highly conserved residues are shown in bold, and the position of residues in the entrance, middle, and bottom of the allosteric binding pocket indicated. Data extracted from [28, 77, 78, 80]

Studies combining structural modelling of the giant panda P2X7 receptor, ligand docking, site-directed mutagenesis, and functional characterisation provide evidence to suggest that this allosteric binding pocket also underpins the specific and potent inhibition by AZ10606120, A74003, and A438079 of the human P2X7 receptor [77, 78]. Introduction of point mutations of residues in the entrance (Lys^110^, Val^304^, and Glu^305^), middle (Phe^88^, Asp^92^, Met^105^, Phe^108^, Lys^297^, Tyr^298^, Ile^310^, and Val^312^), and bottom (Thr^90^, Ala^91^, Tyr^93^, Thr^94^, Phe^95^, Pro^96^, Leu^97^, and Phe^103^) of the above-described allosteric antagonist-binding pocket in the human P2X7 receptor reduced (F88A, T90V, D92A, Y93A, T94V, F103A, and M105A) or increased (K110Y, K297G, V304C, and E305A) the sensitivity to AZ10606120 (Table 1) [77]. It is noted that introduction of F88A, M105A, K110Y, or E305A mutation in the giant panda P2X7 receptor resulted in no change in, whereas V312A mutation in the human P2X7 receptor was with no significant effect on, the sensitivity to AZ10606120 (Table 1). It remains to be confirmed whether such differences reflect the structural differences in the interactions of AZ10606120 with the allosteric binding pocket in the giant panda and human P2X7 receptors. Introduction of above-described mutations in the allosteric binding pocket caused a reduction (F88A, T90V, A91C, D92A, T94V, F95A, P96A, L97P, F103A, M105A, F108C, Y298A, and V312A) or increase (K297G) in the sensitivity to A740003 (Table 1) [77]. These results indicate some differences in mutational effects (F95A and F108C on the sensitivity of the giant panda and human P2X7 receptors to A740003) (Table 1). Introduction of such single mutations also resulted in a reduction (F88A, T90V, A91C, D92A, Y93A, T94V, F95A, P96A, F103A, F108C, Y298A, I310A, and V312A) or increase (L97P, K297G, V304C, and E305A) in the sensitivity to A438079 (Table 1) [77].

There is also evidence to suggest that AZ11645373, KN-62, SB203580, ZINC58368839, calmidazolium, and brilliant blue G (BBG) bind to this same allosteric binding pocket in the human P2X7 receptor. All these antagonists are known to preferentially inhibit the human P2X7 receptor, except BBG which antagonises both the human and rat P2X7 receptors. Structural modelling of the human and rat P2X7 receptors on the zebrafish P2X4 receptor and subsequent molecular docking suggest that of AZ11645373, KN-62, and SB203580 bind to a pocket in close proximity to the ATP-binding site in the human P2X7 receptor [79]. This antagonist-binding pocket is located at the interface of two adjacent subunits in the close vicinity of the α_2_-helix and β_13_ and β_14_ in the upper body, similar to the allosteric binding pocket described in the giant panda P2X7 receptor [28]. More specifically, several residues were predicted to contact, or influence the orientation of, the docked antagonists. Particularly, Phe^95^ in the human P2X7 receptor forms pi-stacking interactions with aromatic rings in antagonists, which is lost in the rat P2X7 receptor containing Leu^95^, explaining the results that replacement of Phe^95^ in the human P2X7 receptor with Leu^95^ in the rat P2X7 receptor reduced the sensitivity to KN-62 [45] and AZ11645373 [80]. Leucine is also present in the guinea pig P2X7 receptor that shows a comparable KN-62 sensitivity to the human P2X7 receptor, indicating that other residues are importantly involved in determining the species-dependent sensitivity of KN-62. The approach combining structural modelling, molecular locking, mutagenesis, and functional characterisation described above to elucidate the binding site for AZ10606120, A74003, and A438079 has been recently applied to examine the interactions of AZ11645373, ZINC58368839, KN-62, calmidazolium, and BBG with the human and rat P2X7 receptors [80]. Single mutation of residues in the allosteric binding pocket discussed above led to strong loss (T94V, F95A, and P96A), reduction (F88A, T90V, A91C, D92A, Y93A, L97P, F103A, F108C, I310A, and V312A), or increase (K110Y) in the sensitivity of the human P2X7 receptor to AZ11645373 (Table 1) [80]. The study further examined the human P2X7 receptor carrying substitution of Ser^86^ at the entrance, Phe^108^ and Val^312^ in the middle, and Phe^95^ in the bottom of the allosteric binding pocket with the equivalent Gly^86^, Tyr^108^, Ala^312^, and Leu^95^ in the rat P2X7 receptor, and also the rat P2X7 receptor harbouring reciprocal mutations. While reciprocal mutations of positions 86 and 108 resulted in no significant effect, the F95L or V312A mutant human P2X7 receptor was ~ 250-fold and ~ 20-fold less sensitive to AZ11645373 and, conversely, the L95F or A312V mutant rat P2X7 receptor exhibited an AZ11645373 sensitivity similar to the wide-type human P2X7 receptor. These results support that residues at positions 95 and 312 are critical in determining the difference in AZ11645373 sensitivity of the human and rat P2X7 receptors. ZINC58368839, identified by virtual screening of the ZINC chemical database against the extended ATP-binding pocket in the human P2X7 receptor structural model generated using the zebrafish P2X4 receptor, antagonises the human P2X7 receptor with a potency of micromolar concentrations [81]. ZINC58368839 has a similar size and is predicted to occupy the same allosteric binding pocket as AZ11645373. The human P2X7 receptor with D92A or F103A mutation became insensitive to ZINC58368839. KN-62, calmidazolium, and BBG are approximately twice as large as AZ11645373 but, nonetheless, can be docked into the same allosteric pocket in the human P2X7 receptor. Consistently, introduction of T90V, D92A, F103A, or V312A mutations in the human P2X7 receptor largely abolished or strongly attenuated the sensitivity to KN-62, calmidazolium, and BBG [80].

In summary, structural and functional studies provide evidence that consistently supports a common inter-subunit allosteric binding pocket for several chemically diverse P2X7 receptor antagonists. The size of this pocket and interactions with residues lining the pocket are critical in determining the specific and high affinity action of these antagonists. The differences in antagonist-interacting residues contribute to the structural basis underpinning species-dependent antagonist sensitivity of P2X7 receptors.

## Concluding remarks

Over the past, more than two decades since molecular identification of the P2X7 receptor, remarkable progress has been made in our understanding of the receptor structure–function relationships, as well as its role in mediating ATP-induced purinergic signalling in diverse physiological and pathological processes. In particular, recent breakthroughs in structural biology have allowed revisiting of earlier mutagenesis and functional studies and facilitated further studies. As discussed above, combined efforts have led to important insights into the structural basis for the functional properties of the P2X7 receptor, including ATP binding, ion permeation, and mechanism of action by chemically diverse P2X7 receptor antagonists. The P2X7 receptor, despite disappointing outcomes from early clinical testing of P2X7 receptor antagonists for the treatment of rheumatoid arthritis, remains an attractive therapeutic target, with promising potential in the development of therapeutics for a diversity of debilitating conditions. Evidently, increased understanding of the structure–function relationships of the P2X7 receptor offers an exciting opportunity to develop structure-guided approaches to develop P2X7-specific antagonists, particularly negative allosteric modulators to tune down the elevated expression and activity of the P2X7 receptor that are often causatively associated with pathological conditions. It is worth pointing out that more efforts are required to provide a full understanding at the molecular level of the unique functional properties of the P2X7 receptors.

## Data Availability

This article does not contain unpublished data, and all data discussed in this article are available in cited publications.
